# The Co-Occurrence of Pertrochanteric Fracture and Acute Coronary Syndrome in a Geriatric Patient: A Case Report and Review of the Literature

**DOI:** 10.3390/jcdd13030132

**Published:** 2026-03-11

**Authors:** Jozef Dodulík, Jiří Demel, Jan Mrózek, Jiří Vrtal, Jiří Plášek, Jan Václavík

**Affiliations:** 1 Department of Internal Medicine and Cardiology, University Hospital Ostrava, 70800 Ostrava, Czech Republic; 2 Department of Internal Medicine, Faculty of Medicine, University of Ostrava, 70300 Ostrava, Czech Republic; 3 Department of Trauma Surgery and Orthopedics, University Hospital Ostrava, 70800 Ostrava, Czech Republic; 4 Institute of Disaster Medicine, Faculty of Medicine, University of Ostrava, 70300 Ostrava, Czech Republic

**Keywords:** geriatrics, STEMI, pertrochanteric femoral fracture, dual antiplatelet therapy, multidisciplinary decision-making, conservative fracture management, skeletal traction

## Abstract

Background: Managing elderly patients with simultaneous acute cardiovascular and orthopedic emergencies presents a unique challenge. While ST-elevation myocardial infarction (STEMI) requires prompt revascularization and dual antiplatelet therapy (DAPT), pertrochanteric femoral fractures usually necessitate early surgical fixation to reduce morbidity and mortality. However, the combination of these conditions complicates both standard treatment pathways. Case presentation: We present the case of an 86-year-old woman admitted after a low-energy fall, with a radiologically confirmed unstable pertrochanteric fracture of the right femur (AO/OTA 31-A2). Upon routine electrocardiogram, anterior STEMI with new-onset atrial fibrillation was diagnosed. Although asymptomatic from a cardiac perspective, bedside echocardiography revealed a severely reduced left ventricular ejection fraction of 10%. Urgent coronary angiography demonstrated a critical mid-left anterior descending lesion, successfully treated with rotational atherectomy, intravascular lithotripsy, and stent implantation. She was initiated on DAPT (aspirin + clopidogrel) and anticoagulated with low-molecular-weight heparin. Given the extremely high bleeding risk, surgical intervention for the femoral fracture was deemed unsafe. Instead, conservative management with skeletal traction (6 kg) was employed. Despite optimal supportive care and early rehabilitation, the patient experienced a complicated hospital course, including delirium, hematuria, and lower respiratory tract infection. She passed away 52 days post-admission. Conclusions: This case illustrates the complexity of clinical decision-making in geriatric patients with competing acute conditions. Current evidence on how to proceed in patients requiring both antithrombotic therapy and urgent orthopedic surgery is limited. Multidisciplinary teams must carefully weigh the risks and benefits of both surgical and conservative strategies. Further guidelines addressing such scenarios in elderly patients are urgently needed.

## 1. Introduction

Pertrochanteric femoral fractures and acute coronary syndromes (ACS) are both major causes of morbidity and mortality in the elderly population, and their co-occurrence represents a rare but particularly complex clinical scenario. The incidence of hip fractures continues to rise globally with the aging population, with an estimated 6.26 million hip fractures expected annually by 2050 [1]. Among these, pertrochanteric fractures (classified as AO/OTA 31-A1 to A3) are associated with significant short- and long-term mortality, particularly in patients with comorbidities or delayed surgical intervention [2,3,4]. Early operative management, often within 48 h when feasible, is associated with reduced mortality and improved functional outcomes [2,3].

Concurrently, ACS remains one of the leading causes of hospitalization and death in the elderly, with approximately one-third of all hospitalized patients with ACS being over the age of 75 [5]. Despite advances in pharmacologic and interventional therapies, older adults with ACS have double the mortality risk of younger cohorts [5]. Treatment decisions are often complicated by polypharmacy, frailty, and a heightened susceptibility to bleeding [5].

Dual antiplatelet therapy (DAPT), typically comprising aspirin and a P2Y12 receptor inhibitor, is essential after percutaneous coronary intervention (PCI) in the context of ACS [6]. However, in patients requiring orthopedic surgery, DAPT significantly increases perioperative bleeding risk and challenges surgical timing [6,7]. On the other hand, premature discontinuation of DAPT in the early post-PCI phase carries a high risk of stent thrombosis, which is associated with substantial mortality [6,7]. In cases where surgery is contraindicated due to bleeding risk or hemodynamic instability, conservative management of hip fractures may be considered. However, this strategy is associated with a considerably worse prognosis. Nonoperative management has been linked to very high 30-day mortality (reported as high as 87% in highly selected frail cohorts) [8,9], primarily due to complications such as pneumonia, pressure ulcers, thromboembolism, and prolonged immobilization.

This report presents the case of an 86-year-old woman with simultaneous anterior ST-elevation myocardial infarction (STEMI), new-onset atrial fibrillation (AF), and an unstable pertrochanteric fracture. We discuss diagnostic and therapeutic challenges, highlight key decision-making dilemmas, and review the relevant literature to contextualize management options for this complex overlap of cardiac and orthopedic emergencies in geriatric care.

## 2. Case Presentation

An 86-year-old female patient was admitted to the emergency department after a domestic fall with direct impact on the right hip. Before the injury, the patient lived alone and was relatively independent in activities of daily living, with support from her family. She was ambulatory using a single cane due to chronic lumbar spine pain. Her medical history included coronary artery disease, prior ischemic stroke, chronic kidney disease, arterial hypertension, diabetes mellitus, dyslipidemia, hypothyroidism on replacement therapy, hyperuricemia, degenerative lumbar spine disease, and vitamin D deficiency. Long-term medication included metformin, a statin, bisoprolol, antihypertensive therapy and other chronic medication.

She was brought in by emergency services primarily due to her inability to bear weight and marked pain localized to the right groin. On admission, she was alert, apparently hemodynamically stable (BP 145/70 mmHg, HR 98/min), afebrile, and without neurologic deficit. Physical examination revealed external rotation, shortening and tenderness of the right lower extremity.

**Figure 1:**
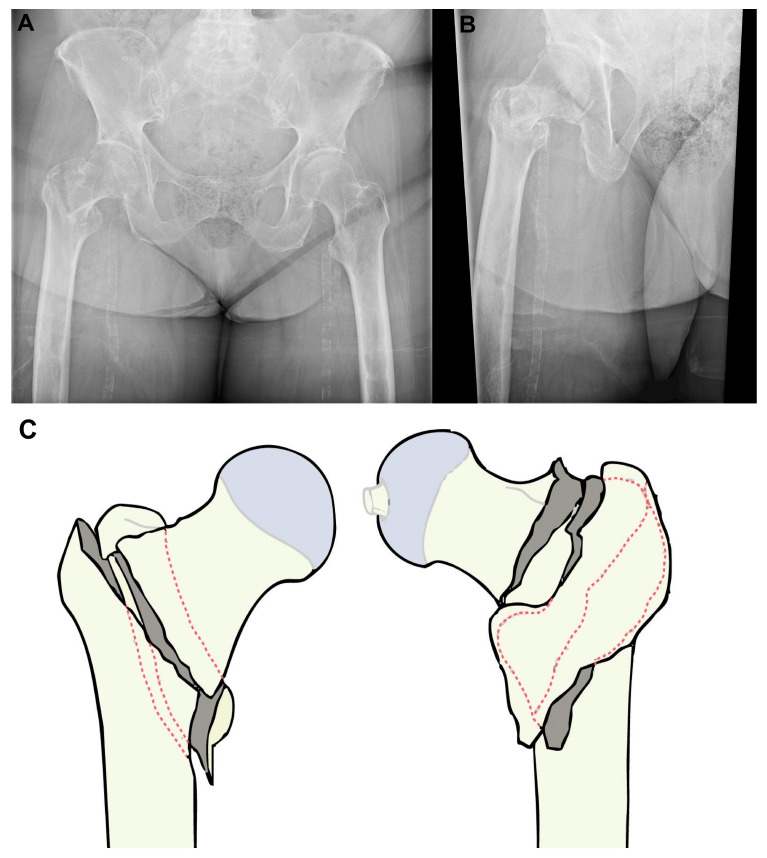
(**A**) Initial pelvic X-ray showing an unstable pertrochanteric fracture of the right femur (AO/OTA 31-A2). (**B**) Control pelvic X-ray demonstrating maintained alignment under traction. (**C**) Schematic illustration of the fracture pattern.

A pelvic X-ray revealed an unstable pertrochanteric femoral fracture (AO/OTA 31-A2) with posteromedial comminution and medial displacement of the femoral shaft (Figure 1A). Orthopedic management initially considered surgical fixation via intramedullary nailing.

**Figure 2:**
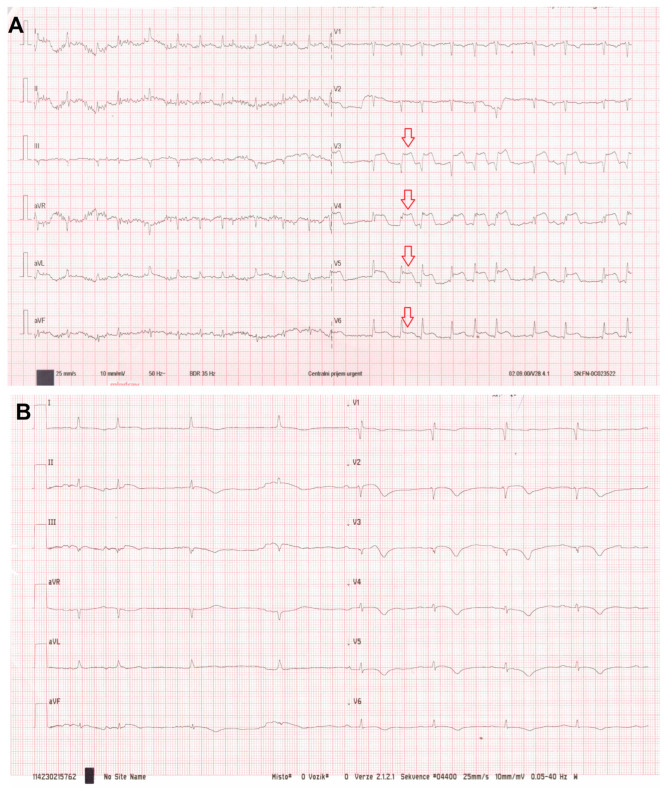
(**A**) The 12-lead electrocardiogram (ECG) showing new-onset atrial fibrillation with rapid ventricular response (~120 bpm) and 3–4 mm ST-segment elevation in leads V3–V6 (arrows). (**B**) The ECG after pharmacological conversion to sinus rhythm.

As part of a routine preoperative assessment, a standard 12-lead electrocardiogram (ECG) was performed, revealing new-onset AF with rapid ventricular response (~120 bpm) and 3–4 mm ST-segment elevations in leads V3–V6 (Figure 2A). From her emergency department arrival to transfer to the catheterization laboratory, including initial vitals and blood sampling, pelvic X-ray, trauma assessment, ECG recognition of STEMI, bedside echocardiography, and multidisciplinary decision making, approximately 100 min elapsed. Notably, the patient denied any chest pain, dyspnea, palpitations or other ischemic symptoms.

**Figure 3:**
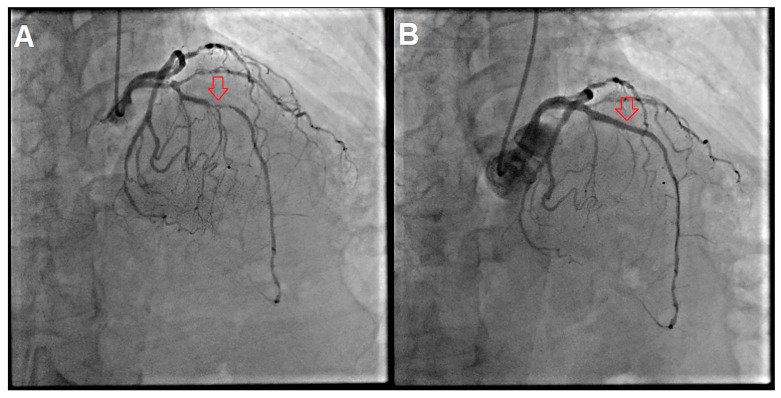
(**A**) Emergent coronary angiography revealed critical stenosis of the mid-left anterior descending artery (LAD) (arrow), significant stenoses of the first diagonal and obtuse marginal branches, a borderline lesion in the circumflex artery, and a hypoplastic right coronary artery. (**B**) Percutaneous coronary intervention was immediately performed with rotational atherectomy, intravascular lithotripsy, and deployment of two drug-eluting stents in the LAD (arrow).

Transthoracic echocardiography performed at the bedside showed a severely reduced left ventricular ejection fraction (LVEF ~10% in the acute phase) and global hypokinesis. Immediately prior to coronary angiography (CAG), her blood pressure was 140/90 mmHg with tachycardia 110–120/min, and admission lactate was 11.3 mmol/L. Despite preserved blood pressure, this marked hyperlactatemia was interpreted as a marker of occult hypoperfusion in the setting of severe acute LV dysfunction and tachyarrhythmia. Emergent CAG revealed critical stenosis of the mid-left anterior descending artery (LAD), significant stenoses of the first diagonal and obtuse marginal branches, a borderline lesion in the circumflex artery, and a hypoplastic right coronary artery (Figure 3A). PCI was immediately performed with rotational atherectomy, intravascular lithotripsy, and deployment of two drug-eluting stents (DESs) in the LAD (Figure 3B). The procedure was performed under analgesia with a fentanyl 100 µg i.v., with the administration of 500 mL of i.v. crystalloid and transient low-dose norepinephrine support; the total contrast volume was 140 mL. No mechanical circulatory support was required. DAPT was initiated immediately after PCI with loading doses administered according to the institutional STEMI protocol (aspirin 300 mg and clopidogrel 600 mg), followed by maintenance therapy (aspirin 100 mg once daily and clopidogrel 75 mg once daily). Given AF of unknown duration and high thromboembolic risk, anticoagulation with therapeutic-dose low-molecular-weight heparin (LMWH) was started: nadroparin/Fraxiparine 86 anti-Xa IU/kg subcutaneously every 12 h; ~5160 anti-Xa IU q12h for 60 kg, adjusted to renal function. Therapeutic-dose LMWH was maintained during the acute phase and subsequently adjusted according to bleeding risk, renal function, and the evolving clinical course. A parenteral, rapidly modifiable anticoagulation strategy was preferred during hospitalization to allow prompt adjustment in case of bleeding or a need for urgent procedures, while renal function was closely monitored.

Post-intervention, high-sensitivity troponin I (hsTnI) peaked at 19,801.7 ng/L. C-reactive protein (CRP) reached 119.2 mg/L with leukocytosis of 21.7 × 10^9^/L. Renal function declined from baseline plasma creatinine 103 to 200 µmol/L during hospitalization. Liver enzymes were elevated: ALT 6.65 μkat/L (~399 U/L) and AST 6.69 μkat/L (~401 U/L). Procalcitonin was negative.

Given the need for urgent hip fracture surgery but the prohibitive bleeding risk associated with recent PCI and combined DAPT + therapeutic-dose anticoagulation, a multidisciplinary team (cardiology, traumatology, and anesthesiology) decided to proceed with non-operative management. The anesthesiology assessment concluded that, given the patient’s acute clinical condition and severe cardiovascular instability risk, general anesthesia was not acceptable at that time; consequently, operative fixation, including limited damage-control procedures, was not pursued. Skeletal traction was applied using a supracondylar femoral pin and a 6 kg weight under adequate analgesia.

Amiodarone was administered intravenously, resulting in pharmacological conversion to sinus rhythm (Figure 2B). The patient was monitored in a high-dependency unit, with measures for pressure ulcer prevention, prevention of immobility related complications, and respiratory support.

After stabilization of the acute cardiac event and delirium, the patient was transferred on day 20 to a long-term care hospital (LTCH) for structured nursing care and bed-based rehabilitation, with ongoing cardiology and traumatology follow-up. In our healthcare setting, a LTCH represents an active post-acute rehabilitation pathway for frail patients requiring prolonged nursing care (including traction management), rather than hospice care. A control radiograph (Figure 1B) confirmed maintenance of fracture alignment.

At outpatient cardiology and orthopedic follow-up, the patient remained hemodynamically stable with no re-elevation of biomarkers. However, her overall frailty progressed, complicated by hypoactive delirium, macroscopic hematuria (non-infectious, self-limited), and respiratory tract infection. She died 52 days after the initial admission due to progressive respiratory failure.

## 3. Discussion

This case highlights the complex decision-making process when two life-threatening conditions, acute myocardial infarction (MI) and unstable hip fracture, coexist in a frail elderly patient. Both conditions independently carry high morbidity and mortality and require divergent and often mutually exclusive treatment strategies. While STEMI generally necessitates urgent reperfusion and DAPT, management of an unstable hip fracture in elderly patients typically favors early surgical fixation (often within 24–48 h when feasible) to minimize complications such as thromboembolism, pneumonia, and prolonged immobility [2,3].

Although chest pain remains the hallmark symptom of MI, elderly patients, especially those with diabetes mellitus and prior cerebrovascular disease, often present with atypical or silent ischemia [5]. In our patient, STEMI was incidentally diagnosed during a routine preoperative ECG, despite the absence of angina, dyspnea, or syncope. Several studies have shown that asymptomatic MIs are not benign; in fact, silent STEMIs carry a prognosis comparable to symptomatic events, particularly in patients with hemodynamic compromise or severely reduced LVEF [5,6]. In such cases, particularly in the presence of the hemodynamic compromise of severely reduced LVEF, urgent CAG and revascularization are generally recommended, even when classic ischemic symptoms are absent [6]. In our patient, bedside echocardiography revealed an LVEF of approximately 10%, which by itself conferred a high risk of adverse outcomes. The European Society of Cardiology (ESC) guidelines strongly recommend prompt PCI in patients with STEMI and signs of cardiogenic shock or severe LV dysfunction, even in the absence of chest pain [6]. The successful PCI with restoration of sinus rhythm after amiodarone therapy suggests that the myocardial insult was indeed recent and hemodynamically relevant.

Pertrochanteric fractures, especially AO/OTA 31-A2 types, are biomechanically unstable and associated with high mortality if left untreated surgically [8]. The standard of care recommends surgical fixation, which allows for early mobilization and reduces systemic complications. However, the presence of DAPT and therapeutic anticoagulation introduces a prohibitive bleeding risk. Current guidelines generally recommend delaying elective non-cardiac surgery after PCI with DES when possible, particularly during the early period when uninterrupted antiplatelet therapy is most critical [7]. Bridging with LMWH does not eliminate bleeding risk and may further increase it in elderly, frail patients. Multiple case series and retrospective studies have evaluated hip fracture management in patients on recent DAPT. Their data are inconsistent: some favor delaying surgery, while others advocate for early surgical intervention under regional anesthesia with meticulous perioperative planning and multidisciplinary input [3,7]. However, there is no high-level evidence to guide management in cases of simultaneous STEMI and unstable orthopedic injury. In our case, after multidisciplinary consensus among cardiologists, anesthesiologists, and traumatologists, a conservative approach with skeletal traction was chosen. This decision was supported by extreme perioperative risk (recent PCI, DAPT with therapeutic-dose anticoagulation and severe LV dysfunction) and the patient’s pre-existing frailty. Although traction does not provide definitive fracture stabilization, it may offer pain control and partial functional alignment, buying time for recovery or palliation [8,9].

To support clinical decision-making in similar scenarios, Table 1 summarizes practical management strategies reported for hip fracture patients requiring recent PCI/DAPT and/or anticoagulation. This table contrasts the timing of surgery, antithrombotic handling, and anticipated trade-offs.

The Clinical Frailty Scale (CFS), which corresponded to grade 6–7 in our patient, is a validated predictor of postoperative mortality and institutionalization [10]. Importantly, in our healthcare system, transfer to a LTCH represents an active post-acute rehabilitation and nursing pathway rather than end-of-life hospice care. Advanced frailty increases the risk of both ischemic and bleeding events, complicates rehabilitation, and impairs response to medical interventions. Delirium developed early in the hospital course, likely due to a combination of systemic inflammation, polypharmacy (including opioids and anticholinergics), and immobility. Delirium in elderly patients with hip fractures is associated with a 2- to 3-fold increase in mortality [11]. Hematuria and recurrent infections added further complexity and likely contributed to the fatal outcome. Despite successful revascularization and rhythm control, the patient’s condition deteriorated in the context of immobility, infectious complications, and the inability to proceed with surgical fixation.

This case underscores the need for individualized, multidisciplinary decision-making in geriatric patients with concurrent cardiovascular and orthopedic emergencies. While ESC and ACC/AHA guidelines provide frameworks for managing STEMI and perioperative antithrombotic therapy, they do not specifically address scenarios involving urgent orthopedic injuries [6,7]. Similarly, orthopedic guidelines do not offer recommendations for patients recently undergoing PCI or requiring DAPT. Our case suggests that conservative management may be a reasonable, albeit imperfect, option in selected frail patients with high hemorrhagic and ischemic risk. Shared decision-making, prognostication, and alignment with patient goals of care are crucial. Finally, this case also raises ethical and logistical questions surrounding goals of care in elderly patients with multiple comorbidities. The initial favorable hemodynamic status and successful PCI created the illusion of physiological reserve, yet the inability to proceed with definitive orthopedic repair due to antithrombotic therapy ultimately led to functional decline and death. Future guidelines should consider providing structured algorithms or flowcharts for perioperative antithrombotic management in elderly patients with competing acute indications for surgery and DAPT. Further research is warranted to evaluate outcomes of conservative versus surgical approaches in this growing patient population.

## 4. Conclusions

This case illustrates the challenging intersection between acute MI requiring immediate PCI and DAPT, and an unstable hip fracture requiring timely surgical fixation. In elderly, frail patients, such dual emergencies often lack clear evidence-based pathways and necessitate nuanced, multidisciplinary decisions. Our patient, initially asymptomatic from a cardiological perspective, was diagnosed with anterior STEMI during evaluation for traumatic injury. Despite successful PCI and rhythm control, orthopedic surgery was deferred due to excessive hemorrhagic risk, and the fracture was managed conservatively with traction. Prolonged immobility and frailty contributed to complications including delirium, hematuria, infection, and ultimately death. This case highlights the need for individualized management, integration of frailty assessment, and calls for future guideline development to address dual-priority emergencies in geriatric care.

## Figures and Tables

**Table 1:** Practical strategies for managing unstable hip fracture in frail patients after recent PCI requiring DAPT and/or therapeutic anticoagulation.

Strategy	When Considered	Antithrombotic Approach (Typical)	Key Trade-Offs/Expected Issues
Early surgery (≤48 h) with multidisciplinary planning	If bleeding risk acceptable and surgery unavoidable	Continue aspirin, evaluate P2Y12 interruption vs. continuation; prefer regional anesthesia when feasible; resume P2Y12 early post-op	Lower immobilization complications; higher perioperative bleeding risk
Delayed surgery (days–weeks)	If ischemic risk mandates DAPT continuation (early post-DES)	Continue full DAPT initially; surgery postponed until safer window; reassess daily	Reduces early bleeding risk; increases risk of delirium, infections, thromboembolism, deconditioning
Conservative management with traction	If perioperative risk prohibitive (severe LV dysfunction, frailty, active complications)	Maintain DAPT ± therapeutic anticoagulation as indicated; focus on analgesia, nursing care, bed-based rehab	Avoids surgical bleeding; high risk of immobility-related complications and functional decline
Damage-control/limited fixation (selected)	If partial stabilization is needed but full surgery too risky	Individualized: minimal interruption of antiplatelets; close hemostasis strategy	Potential compromise between bleeding and mobilization; evidence limited

## Data Availability

All data supporting the findings of this case report are contained within the manuscript. Additional anonymized clinical data, imaging, and laboratory results can be provided by the corresponding author upon reasonable request and in accordance with institutional policies.

## References

[1] Cooper C., Campion G., Melton L.J. (1992). Hip fractures in the elderly: A world-wide projection. Osteoporos. Int..

[2] Seong Y.J., Shin W.C., Moon N.H., Suh K.T. (2020). Timing of Hip-fracture Surgery in Elderly Patients: Literature Review and Recommendations. Hip Pelvis.

[3] O’Connor M.I., Switzer J.A. (2022). AAOS Clinical Practice Guideline Summary: Management of Hip Fractures in Older Adults. J. Am. Acad. Orthop. Surg..

[4] Andaloro S., Cacciatore S., Risoli A., Comodo R.M., Brancaccio V., Calvani R., Giusti S., Schlögl M., D’angelo E., Tosato M. (2025). Hip Fracture as a Systemic Disease in Older Adults: A Narrative Review on Multisystem Implications and Management. Med. Sci..

[5] Engberding N., Wenger N.K. (2017). Acute Coronary Syndromes in the Elderly. F1000Research.

[6] Byrne R.A., Rossello X., Coughlan J.J., Barbato E., Berry C., Chieffo A., Claeys M.J., Dan G.-A., Dweck M.R., Galbraith M. (2023). 2023 ESC Guidelines for the management of acute coronary syndromes: Developed by the task force on the management of acute coronary syndromes of the European Society of Cardiology (ESC). Eur. Heart J..

[7] Halvorsen S., Mehilli J., Cassese S., Hall T.S., Abdelhamid M., Barbato E., De Hert S., de Laval I., Geisler T., Hinterbuchner L. (2022). 2022 ESC Guidelines on cardiovascular assessment and management of patients undergoing non-cardiac surgery. Eur. Heart J..

[8] Bui M., Groothuis-Oudshoorn C.G.M., Witteveen A., Hegeman J.H. (2024). Palliative Non-Operative Management in Geriatric Hip Fracture Patients: When Would Surgeons Abstain from Surgery?. J. Clin. Med..

[9] Wijnen H.H., Schmitz P.P., Es-Safraouy H., Roovers L.A., Taekema D.G., Van Susante J.L.C. (2021). Nonoperative management of hip fractures in very frail elderly patients may lead to a predictable short survival as part of advance care planning. Acta Orthop..

[10] Rockwood K., Song X., MacKnight C., Bergman H., Hogan D.B., McDowell I., Mitnitski A. (2005). A global clinical measure of fitness and frailty in elderly people. CMAJ.

[11] Witlox J., Eurelings L.S.M., de Jonghe J.F.M., Kalisvaart K.J., Eikelenboom P., van Gool W.A. (2010). Delirium in Elderly Patients and the Risk of Postdischarge Mortality, Institutionalization, and Dementia. JAMA.

